# Microscopic dissipative structuring and proliferation at the origin of life

**DOI:** 10.1016/j.heliyon.2017.e00424

**Published:** 2017-10-13

**Authors:** Karo Michaelian

**Affiliations:** Department of Nuclear Physics and Application of Radiations, Instituto de Física, Universidad Nacional Autónoma de México, Apartado Postal 20–364, Cuidad de México, Mexico

**Keywords:** Thermodynamics, Statistical physics, Quantum mechanics, Microbiology, Evolution, Biochemistry

## Abstract

Some fundamental molecules of life are suggested to have been formed, proliferated, and evolved through photochemical microscopic dissipative structuring and autocatalytic proliferation under the UV-C/UV-B solar environment prevalent at Earth’s surface throughout the Archean. Evidence is given in the numerous salient characteristics of these, including their strong absorption in this spectral region and their rapid non-radiative excited state decay through inherent conical intersections. The examples of the dissipative structuring and dissipative proliferation of the purines and of single strand DNA are given. UV-C and UV-B-induced stationary state isomerizations and tautomerizations are shown to be crucial to the formation of the purines from hydrogen cyanide in an aqueous environment under UV-C light, while UV-C induced phosphorylation of nucleosides and denaturing of double helix RNA and DNA are similarly important to the production and proliferation of single strand DNA. This thermodynamic dissipation perspective provides a physical-chemical foundation for understanding the origin and evolution of life.

## 1 Introduction

It is well known empirically, and described theoretically from within the framework of Non-linear Classical Irreversible Thermodynamics (Prigogine, 1967), that “spontaneous” macroscopic organization of material in space and time can occur within a system subjected to an externally imposed generalized chemical potential. This arises from a non-equilibrium thermodynamic imperative to increase entropy production, i.e. to increases in the spread of the conserved quantities of Nature (energy, momentum, angular momentum, charge, etc.) over ever more microscopic degrees of freedom. Ilya Prigogine’s analysis of the phenomenon of “spontaneous” dissipative structuring of material under a generalized chemical potential garnered him the 1977 Nobel Prize in chemistry. Almost a century earlier, however, Boltzmann (1886) had already realized that organic material was “struggling” (in a Darwinian sense) to dissipate the impressed solar photon potential and it was Schrödinger (1944) who is credited with bringing Boltzmann’s deep insight to the forefront of scientific investigation on the physics and chemistry of life.

The existence of non-linearity between the generalized flows and forces derived from an impressed external potential will endow the material in the system with numerous possible solutions for the time-relaxed non-equilibrium stationary state. These solutions often correspond to stable but dynamic material organizations or processes of low entropy but high entropy production, involving symmetry breaking in both space and time (material structuring). Which stable solution the system evolves into depends on the initial conditions and on a particular random microscopic fluctuation at a bifurcation point giving rise to the different solutions. This endows the physical-chemical process with a particular evolutionary history, generally through states of greater global entropy production.

Common examples of dissipative structuring occurring in systems held out of equilibrium by an external generalized chemical potential are space-symmetry breaking convection cells and time- and space-symmetry breaking patterns of a Belousov-Zhabotinsky chemical oscillator. The former arise in conduction-convection systems held under gravity at an imposed temperature gradient, while the latter arise in diffusion-reaction systems under imposed chemical affinities. Structuring in both conduction-convection and diffusion-reaction systems have an inferior size limit due to characteristic finite diffusion lengths. For biological systems in a water solvent environment, the minimum size of dissipative structures of the conduction-convection or reaction-diffusion type is of the order of microns (Hess and Mikhailov, 1994).

However, dissipative structuring occurs at the sub-micron, and even at the nanometer, scale as witnessed by the many processes occurring within a living cell. An example being molecular motors, such as those promoting the advance of polymerase during the extension of RNA or DNA (Bustamante et al., 2005), or the process of the formation of peptide bonds between selected amino acids, known as translation, which occurs in ribosomes of the cell. There must, therefore, exist other dissipative mechanisms besides conduction-convection or reaction-diffusion operating at the microscopic level which drive microscopic irreversible processes and their associated dissipative structuring within cells.

At the nanoscale dimension, particularly for soft (biological) material, strong and directional covalent bonds allow for molecular structural transitions; for example between different isomeric, tautomeric, or charged states of the molecule. There is also the possibility of transitions between the different electronic, vibrational, or rotational states, between different orientations of electric or magnetic dipole moments under an externally imposed electric or magnetic field, or between molecular complexes or excimer states, or even microscopic phase separation (gas–liquid–solid). These transformations are related to the *internal* degrees of freedom of the molecule, or the molecular complex, which are available for microscopic structuring and which can foment the dissipation of the impressed potential.

Chemical or photochemical reactions or diffusion, together with the possible internal molecular structural transitions mentioned above, can lead to nanoscale structuring through the dissipation of a generalized chemical potential. This new field of research, referred to as *microscopic dissipative structuring*, although already treated formally by Prigogine (1967) through the analysis of internal degrees of freedom, is only recently receiving attention due to its potential for application in nanotechnology. The innovation is that dissipation-induced nanoscale structuring can persist even after removal of the external generalized chemical potential, due to atomic and molecular mobility issues resulting from strong inter-atomic interactions, and can thus be used in a large number of novel practical applications where controlled nanostructuring is required (Hess and Mikhailov, 1994; Soejima et al., 2009; Soejima et al., 2014).

Photon-induced processes are often associated with microscopic dissipative structuring since photons can deliver an intense amount of energy of low entropy locally on a very small amount of material. This leads to very large generalized forces at microscopic dimensions which promote microscopic “self-organization” as a thermodynamic response to dissipate the applied photon potential. In particular, quantum resonances can lead to the molecular transformations mentioned above, which, through their dissipative or probabilistic nature of decay, break classical reversibility (Prigogine and Stengers, 1997) by an amount depending on the amount of energy in the photon and the interaction between absorbing atoms involved (Lucia, 2017). Today there are, in fact, many examples of biology utilizing photon-induced molecular dissipative transformations at the quantum level (e.g. Schulten and Hayashi, 2014) and in Section 4 I present examples which may have led to the origin of life.

## 2 Theory

### 2.1 Thermodynamic formalism for treating microscopic dissipative structuring

The non-equilibrium thermodynamic formalism for treating microscopic dissipative structuring involving internal degrees of freedom is similar to that for treating macroscopic dissipative structuring and was first studied by Prigogine (1967). The starting point is the Gibb’s equation,

(1) $$\frac{dS}{dt}=\frac{1}{T}\frac{dE}{dt}+\frac{p}{T}\frac{dV}{dt}-\frac{1}{T}\int_{\gamma }\mu (\gamma )\frac{\partial n(\gamma )}{\partial t}d\gamma$$

where n(γ) is the number of molecules in a state defined by some internal coordinate γ (for example the angle ϑ of the electrical dipole moment of the molecule with respect to an externally applied electric field). Therefore, n(γ)dγ is the number of molecules for which the internal coordinate lies between γ and γ + dγ. Assuming that the change of γ is discrete, i.e. γ can be changed by transformations from, or into, neighboring states γ −1 or γ +1, then the continuity equation is,

(2) $$\frac{dn(\gamma )}{dt}+v_{\gamma }-v_{\gamma -1}=0\text{,}$$

where *v*_γ_ is the rate for the transition γ→(γ+1) and *v*_γ−1_ is the rate for (γ−1)→γ. If γ is a continuous parameter, the equivalent equation is,

(3) $$\frac{\partial n(\gamma )}{\partial t}+\frac{\partial v(\gamma )}{\partial \gamma }=0$$

which is a continuity equation in the internal coordinate γ. *v*(γ) is the reaction rate (flow) giving the net flow of molecules into and out of the state defined by the coordinate γ. In vector notation, Equation (3) becomes

(4) $$\frac{\partial n(\gamma )}{\partial t}-div(v(\gamma ))=0\text{.}$$

By partial integration, Equation (1) can be transformed into

(5) $$\frac{dS}{dt}=\frac{1}{T}\frac{dE}{dt}+\frac{p}{T}\frac{dV}{dt}-\frac{1}{T}\int_{\gamma }\frac{\partial \mu (\gamma )}{\partial \gamma }v(\gamma )d\gamma .$$

The first two terms on the right are the flow of entrop, d_e_S/dt, from or into the external environment due to the flow of energy and work performed on the system, so the internal production of entropy due to the flow along the internal coordinate γ is

(6) $$\frac{d_{i}S}{dt}=-\frac{1}{T}\int_{\gamma }\frac{\partial \mu (\gamma )}{\partial \gamma }v(\gamma )d\gamma >0.$$

One can now postulate a further refinement of the second law of thermodynamics, valid within the microscopic internal coordinate space; in each part of the internal coordinate space, the irreversible processes proceed in a direction such that a positive entropy production results (Prigogine, 1967). This implies

(7) $$\sigma^{*}=-\frac{1}{T}\frac{\partial \mu (\gamma )}{\partial \gamma }v(\gamma )>0$$

where σ* is the entropy production per unit volume of the internal coordinate (or configuration) space. σ* has the usual form of a product of an affinity or force, $X=-\frac{1}{T}\frac{\partial \mu (\gamma )}{d\gamma }$, and a flow or rate, J = *v*(γ), of an irreversible process.

### 2.2 The dissipation-replication relation

Just as in macroscopic non-equilibrium thermodynamics, linear relations between the forces and flows lead to a unique stationary state with a minimum of entropy production (with respect to the organization of the free forces *X*_α_ or flows J_α_; those not fixed by the environment). However, for non-linear relations between forces and flows, multiple stationary states exist, each with a possible different entropy production rate. Which solution is chosen by nature is determined by a microscopic fluctuation at a bifurcation, but it is more probable that fluctuations leading the system to greater internal entropy production will dominate the history of evolution towards new stationary states (Michaelian, 2016).

In the non-linear regime, the evolution of the system is no longer dictated by the principle of minimum entropy production, but rather by the general evolutionary criterion of Glansdorff and Prigogine (1964) which states that the variation of the entropy production, $P=d_{i}S/dt=\sum_{\alpha }X_{\alpha }J_{\alpha }$, due to variation of the free forces is negative semi-definite,

(8) $$\frac{d_{X}P}{dt}=\frac{dP}{dt}-\frac{d_{J}P}{dt}\leq 0,$$

where $d_{X}P/dt$ is the variation of the entropy production with respect to the free forces and $d_{J}P/dt$ is the variation of the entropy production with respect to the free flows. In the stationary state $d_{X}P/dt=0$ and this criterion can be used to show (Michaelian, 2013; see also Prigogine, 1967 for the original case of a purely chemical autocatalytic reaction) that, in the stationary state; 1) any product of a photochemical reaction (for example, a pigment), which acts as a catalyst for the dissipation of the same photon potential that produced it, will increase its concentration many-fold over what would be expected if the system were close to equilibrium or if the product did not act as a catalyst for dissipation, giving rise to a dissipation-replication relation, and 2) that the internal entropy production of the system increases. Given a constant external photon flux and a constant sink of the heat of dissipation, and the dispersal of the photochemical reaction products, this will lead to a continual proliferation of photochemical reaction products (pigments). Recognizing, in this manner, the fundamental molecules of life as autocatalytic microscopic dissipative structures can thereby explain the “vitality” of life at life’s beginnings and, indeed, throughout its evolutive history (Michaelian, 2016). Organic complexes are thus likely selected by nature not on the basis of some indefinable “fitness function”, nor on their photostability (Sagan, 1973; Mulkidjanian et al., 2003), but rather on the basis of their ability to catalyze photon dissipation and thereby secure their proliferation through this thermodynamic dissipation-replication relation.

## 3 Background

### 3.1 The fundamental molecules of life are microscopic dissipative structures

The ubiquity of high energy photons arriving at the surface of planets coupled with the much larger number of photochemical reaction pathways available compared to thermal reactions starting from the electronic ground state, would lead to the expectation that the first molecules of life were photo-chemically produced (Mauzerall, 1990). The non-equilibrium thermodynamic principles outlined in Section 2, and the need for photon energies ≳ 3.0 eV to break and reform carbon covalent bonds, would further suggest that some fundamental molecules of life (common to all three domains of life; archea, bacteria, and eukaryote), and their associations in polymers or other complexes, are examples of microscopic dissipative structuring which occurred under the Archean UV-B and UV-C photon potential (Michaelian, 2009; Michaelian, 2011; Michaelian, 2013; Michaelian and Simeonov, 2015; Michaelian and Simeonov, 2017).

**Fig. 1:**
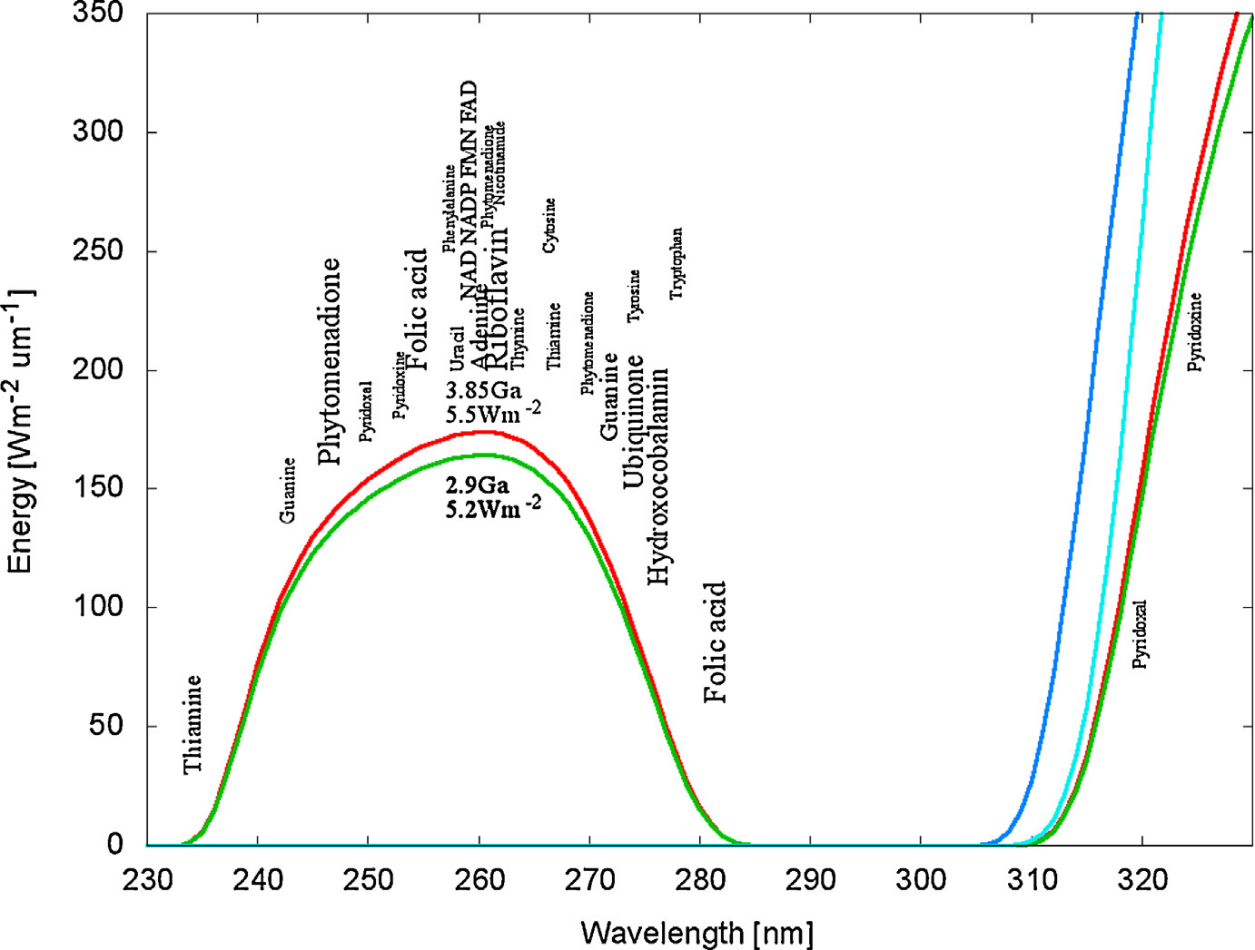
The wavelengths of maximum absorption of many of the fundamental molecules of life (common to all three domains) coincide with a predicted atmospheric window in the UV-C at the time of the origin of life at 3.85 Ga and until at least 2.9 Ga (red and green curves respectively). By 2.2 Ga (light blue curve) the UV-C light at Earth’s surface had been extinguished by the oxygen and ozone resulting from organisms performing oxygenic photosynthesis. The dark blue curve corresponds to the present day surface spectrum. The font size of the letter roughly indicates the relative size of the molar extinction coefficient of the indicated fundamental molecule (pigment). Image credit; adapted from (Michaelian and Simeonov, 2015).

Evidence for this is found in the strong absorption cross sections of many of the fundamental molecules, with maximum absorption coinciding with an atmospheric window for UV-C light existing throughout the Archean (Sagan, 1973, see Fig. 1). Many fundamental molecules also have conical intersections (or have chemical affinity to fundamental molecules having conical intersections) allowing for rapid (picosecond) dissipation of the absorbed photon energy into heat.

Formation and activation process, such as; UV-C induced formation of many fundamental molecules (see below), UV-C induced polymerization of the nucleotides (McReynolds et al., 1971; Szostak, 2001), UV-C induced phosphorylation of amino acids, sugars and lipids, and UV-C induced denaturing of DNA (Michaelian and Santillán Padilla, 2014) also attest to the probable importance of this light to life’s origins.

The nucleobases, as well their polymerization into single and double strand RNA and DNA, efficiently absorb and dissipate UV-C light. The nucleobases can be formed from simpler precursor molecules such as hydrogen cyanide, HCN, by the very same UV-C photons that they dissipate with such efficacy. The photochemical reaction pathways required for constructing many fundamental molecules still remain to be discovered, however, particular instructive examples of the dissipative structuring of the purines and of single strand RNA or DNA are given in the following section.

## 4 Analysis

### 4.1 Microscopic dissipative structuring of the purines from HCN under UV-C Light

The most abundant carbon-containing 3-atom molecule in the observable universe is hydrogen cyanide HCN. Photon disassociation of HCN is observed only at wavelengths < 200 nm (Cicerone and Zellner, 1983) so that once formed, ostensibly through photochemical reactions on N_2_ and CH_4_ (Trainer et al., 2012), in an oxygen-and ozone-less upper atmosphere of early Earth and settling to the surface, it could accumulate without appreciable loss (see Fig. 1).

**Fig. 2:**
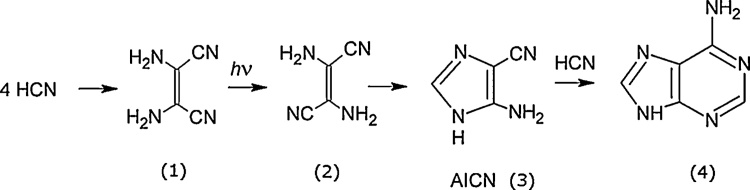
Generic photochemical pathway to the purines first discovered by Ferris and Orgel (1966). Four molecules of HCN are transformed into the smallest stable oligomer (tetramer) of HCN, known as cis-2,3-diaminomaleonitrile (cis-DAMN), (1), which, under a constant UV-C photon flux isomerizes into trans-DAMN (2) (also called diaminofumaronitrile, DAFN) which may be further converted on absorbing two more UV-C photons into an imidazole intermediate, 4-amino-1H-imidazole-5-carbonitrile, AICN (3), which through thermal reaction with another HCN molecule forms a purine, in this case adenine (4). Image credit: Boulanger et al. (2013), adapted from Ferris and Orgel (1966), reprinted with permission.

Oró and Kimball were the first to discover a reaction pathway from HCN to the purine nucleobases (Oró, 1961; Oró and Kimballl, 1962). Later, Saladino et al. (2005) and Saladino et al. (2007) showed that all the nucleobases except guanine could be formed from formamide, H_2_NCOH (a common product of HCN and H_2_O) in a water environment at high temperatures between 100 °C and 160 °C with a number of distinct prebiotically available minerals acting as catalysts. However, and more important to the thesis presented here, Ferris and Orgel (1966) discovered a low temperature generic photochemical pathway to the purines from HCN, as presented in Fig. 2 for adenine. Ferris (2005) also showed how guanine could be obtained from the same basic sequence of photon-induced isomerizations in an aqueous solution with either the aid of a fulminate ion (CNO^−^) or simple hydrolysis. Finally, Barks et al. (2010) have shown that guanine could also be derived from formamide under UV irradiation at low temperatures.

The formation of the nucelobases at high temperature from HCN or formamide and inorganic catalysts as discovered by Oró (1961) and Oró and Kimball (1962) and further investigated by Saladino et al. (2005) and Saladino et al. (2007) may have been operative well before the origin of life at 3.85 Ga, perhaps even during the Hadean (4.6–4.0 Ga) when surface temperatures were well above 100 °C. However, by the origin of life at 3.85 Ga there is evidence that Earth’s surface temperature had cooled to ∼80 °C (Knauth, 1992, Knauth, 2005; Knauth and Lowe, 2003), too cold for normal thermal chemistry with HCN and formamide, and the photochemical pathway to producing the nucleobases (Fig. 2) appears to be the most viable option of high yield so far discovered. A more important reason for emphasizing this photochemical reaction pathway over other routes, however, is that this pathway exemplifies the characteristics of microscopic dissipative structuring and autocatalytic proliferation, which appear to be the relevant hallmarks of life even today.

**Fig. 3:**
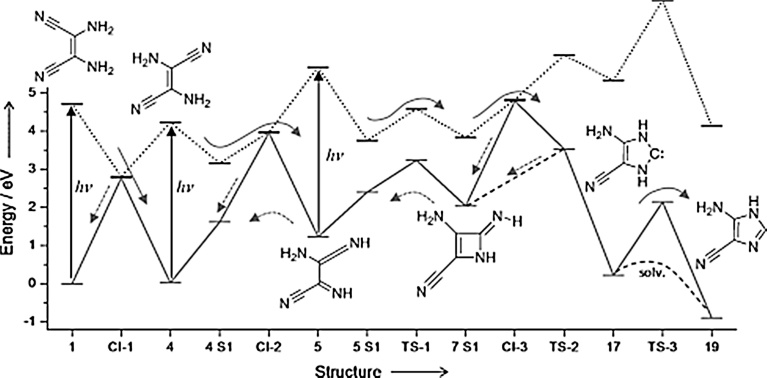
The sequence of photochemical and chemical reactions that lead from cis-DAMN at (1) to trans-DAMN at (4) to AIAC at (5) to AICN at (19). The solid arrows correspond to the forward reactions and the dotted arrows to possible backward reactions. The upward facing arrows indicate where photon absorption (> 4 eV, UV-C) is required for the reaction to proceed. Image credit: Boulanger et al. (2013), reprinted with permission.

The process leading from 4HCN to the tetramer cis-DAMN (1) is exothermic so would occur through normal thermal chemistry, but relatively high free energy barriers imply that local heating would accelerate the process. The reaction steps leading from product (1) through (2) to (3) of Fig. 2 have recently been determined by Boulanger et al. (2013) and Szabla et al. (2014) by analyzing the possible mechanisms of the steps in detail using chemical kinetics and computational chemistry, employing density functional theory and time dependent density functional theory to determine the minima and transition states of the electronic ground state and excited state, respectively. The fast decay of the excess energy due to photon absorption and internal conversion into vibrational energy, with approximately 2/3 of the excess energy being dissipated within 0.2 ps to the surrounding water molecules, indicated that maximum free energy barrier heights for hot ground state thermal reactions are approximately 30 kcal mol^−1^ (Boulanger et al., 2013). Any barriers higher than this would have to be overcome by absorbing a photon. Using such kinetic constraints and transition state formalism, they find that there is only one sequence of steps that is thermodynamically and kinetically compatible with experiment. This sequence is represented schematically in Fig. 3.

**Fig. 4:**
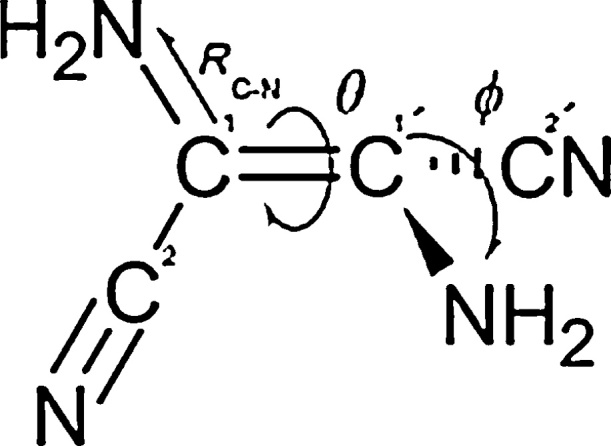
There are in general three internal coordinates involved in the photoisomerization of cis- to trans-DAMN. The first is the rotation angle θ about the C=C double bond, and the second is the pyramidalization coordinate ф of the C1′ carbon atom defined as the angle between the C—N bond and the plane defined by the C1=C1′—C2′ plane, and the third is the elongation of the C—N bond. Image credit: Adapted from Szabla et al. (2014), reprinted with permission.

The full reaction requires the photo-excitation of cis-DAMN (1), trans-DAMN (4), and 2-amino-3-imino acrylamide cyanide AIAC (5), with photons of greater than 4.0 eV (UV-C). Koch and Rodehorst (1974) have found that irradiation with UV-C light induces photoisomerization of cis-DAMN to trans-DAMN with a quantum efficiency of 0.045, leading to a photostationary state with a large predominance of trans-DAMN (4) isomer over cis-DAMN (1) in the ratio of 80:20. This cis-trans isomerization under the UV-C light involves three internal molecular coordinates; rotation around the double C=C bond, the pyramidalization of one of the carbon atoms which stabilizes charge transfer in the photon-induced ^1^ππ* transition (Levine and Martínez, 2007) and the elongation of the C—N bond (see Fig. 4). There are also two main types of cis–trans photoisomerization mechanisms through either singlet or triplet states (Waldeck, 1991), each being competitive in this case (Szabla et al., 2014). The measure of the rotation (angle θ of Fig. 4) amounts to 3.8° and 181.98° for cis-DAMN and trans-DAMN in the ground state, respectively (Szabla et al., 2014). At the MP2 level of optimization, cis-DAMN is the thermodynamically more stable isomer (ΔG_cis–trans_ = 0.61 kcal mol^−1^) owing to less strained steric interaction in the ground state (Szabla et al., 2014).

The wavelength of maximum ^1^ππ* transition was predicted by Szabla et al. (2014) to be 264 and 300 nm for cis-DAMN and trans-DAMN, respectively, with a corresponding red shift when the calculation was carried out in a polar solvent (290 and 326 nm for water). Their calculations show that the minimum energy structure of the bright ^1^ππ* excited state is virtually identical to that of the conical intersection, having dihedral angle θ = 75.2° and prymidalization angle ϕ = 25.8°, which can lead to the cis-trans isomerization.

Without a continuous UV photon flux populating the trans-configuration, the formation of adenine would not occur since there is only a route to it through trans-DAMN which is separated from cis-DAMN by a large energy barrier (see Fig. 3). This isomerization is a clear example of microscopic dissipative structuring involving a continuous photon flux and the internal electronic and isomeric degrees of freedom of the molecule. Another example is the photon-induced tautomerization from trans-DAMN (4) to AIAC (5) corresponding to an amino–imino tautomerization processes mediated by intramolecular proton transfer.

The photoisomerization leading from the stationary state of ratio 20:80 cis-DAMN to trans-DAMN (Fig. 3 (4)) to AICN (Fig. 3 (19)) which requires two UV-C photons to complete, proceeds with a quantum efficiency of 0.0034 (Koch and Rodehorst, 1974). This underscores an important result; although energies of greater than 4.0 eV are added to the system through photoexcitation by UV-C photons, most of this free energy, rather than being consumed in the photochemical reactions, is instead simply dissipated to the ground state of the relevant structure through internal conversion occurring in less than 0.2 ps (Boulanger et al., 2013). cis-DAMN (Fig. 3 (1)) is excited about 300 times (on average) before a single cyclization event (Fig. 3 (19)) takes place (Koch and Rodehorst, 1974).

The final process leading from AICN (Fig. 2 (3)) to Adenine (Fig. 2, (4)) is exothermic although some free energy barriers are quite high (Roy et al., 2007), but still less than the 30 kcal mol^−1^ (1.3 eV) limit determined by Boulanger et al. (2013) for hot ground state reactions. As in the first process leading from 4HCN to the tetramer cis-DAMN (1), here also, heating would accelerate this final reaction set. This could make the reaction autocatalytic. For example, it is known that stacking interactions exist between aromatic rings with their delocalized pi-electrons, but also, and often of greater strength, between an aromatic ring and an aliphatic molecule with localized electrons (Bloom and Wheeler, 2011). It is probable, therefore, that an affinity due to either electrostatic, dispersive, or charge transfer bonding (Gung et al., 2007) exists between AICN (Fig. 2(3)) and adenine (Fig. 2(4)) and therefore resonant energy transfer or the local heat generated through UV-C photon dissipation on an adenine-AICN complex, together with the fact that the formation of Adenine is very exothermic, may accelerate the thermal chemistry leading from 4HCN to adenine, making the overall process autocatalytic. Evidence for this is in the strong temperature dependence of the cis-DAMN to AICN conversion (Becker et al., 1973) and could explain the very high yields (77–82% – Ferris and Orgel, 1966; 100% – Becker et al., 1973) of AICN found in experiments starting with the HCN tetramer cis-DAMN in aqueous solution at room temperature under UV light (240–410 nm). Additionally, water itself acts as a catalyst for the conversion of AICN (Fig. 2(3)) to adenine (Fig. 2(4)) by reducing the size of some of the free energy barriers (Roy et al., 2007).

A similar photochemical route to guanine exists through the hydrolosys of AICN (Ferris, 2005). The photochemical formation of the purines from HCN is thus a very dissipative process and the experimental data presented by Koch and Rodehorst, along with the molecular dynamic simulations of Boulanger et al. (2013) and Szabla et al. (2014), strongly suggest that individual steps in the overall observed process of purine formation from HCN under a UV flux are examples of microscopic dissipative structuring in which, through photon-induced isomerizations and tautomerizations, molecular structures (pigments) arise “spontaneously” to dissipate the same external generalized chemical potential that produced them. As would be expected of dissipative structuring, the final photochemical product, adenine, appears to be “optimized” through this reaction set to strong absorption and dissipation at precisely the wavelength of maximum intensity of the Earth’s Archean surface solar flux in the UV-C (see Table 1 and Fig. 1).

**Table 1:** Steps in the photochemical reaction pathway leading from 4HCN to adenine according to Boulanger et al. (2013) (see Fig. 3). The wavelength at which isomerization to the following step was observed in experiment, λ_isom_, as well as the quantum yield, Φ, for the photochemical conversions is given. Also given is the wavelength of maximum absorption, λ_max,_ of the molecule and the corresponding molar extinction, ε, in an aqueous environment. All referenced values are experimental except (1) and (9) which are calculations at the CASPT2//SA-2-CASSCF(2,2)/cc-pVTZ and (TD)CAM-B3LYP/aug-cc-pVTZ levels respectively.

Step (Fig. 3)	Molecule	λ_isom_ (nm)	Φ	λ_max_ (nm)	ε (M^–1^ cm^−1^)
–	4HCN	thermal			
1	cis-DAMN	254 ^(4)^	0.045 ^(2)^	290 ^(1)^, 298 ^(2)^,	14,000 ^(2)^,
				295 ^(4)^,	12,000 ^(4)^,
				295 ^(8)^	13,500 ^(8)^
4	trans-DAMN	<325 ^(4)^	0.0034 ^(2)^	326 ^(1)^, 313 ^(2)^	8,500 ^(2)^,
				314 ^(4)^, 310 ^(7)^	8,000 ^(5)^
5	AIAC	275 ^(9)^	–	255–290 ^(4)^	–
19	AICN	thermal		250 ^(2)^	10,700 ^(2)^,
				247 ^(3)^	
				245–250 ^(4)^, 246 ^(7)^	11,000 ^(3)^
–	adenine			260 ^(6)^	14,000 ^(6)^
				261 ^(10)^	13,400 ^(10)^
				259 ^(11)^	15,040 ^(11)^

References; (1) Szabla et al., 2014, (2) Koch and Rodehorst, 1974, (3) Ferris and Orgel, 1966, (4) Becker et al., 1973 (22 °C), (5) Sanchez et al., 1967, (6) Clark et al., 1965, (7) Yamada et al., 1968, (8) Ferris and Kuder, 1970, (9) Boulanger et al., 2013, (10) Fasman, 1975, (11) Cavaluzzi and Borer, 2004.

The starting primordial material for the dissipative structuring of the purines may have been formamide rather than (or in addition to) HCN since, as noted at the beginning of this section, similar routes to AICN under UV-C and low temperatures have been discovered using this molecule (Saladino et al., 2007; Barks et al., 2010). Formamide results from the hydrolysis of HCN and the relative yields of adenine obtained through photochemical reactions on the different starting materials depends on the concentration of HCN, greater concentrations favoring the tetramer of HCN over formamide as the starting material (Costanzo et al., 2007).

### 4.2 Nucleoside structuring under UV-C irradiation

Assuming that the nucleobases were formed during the Archean through microscopic dissipative structuring under UV light, as outlined for the purines in this section, it would have been crucial to such a dissipative program to keep the nucleobases at the surface of the ocean where they would be exposed to maximum UV-C light. Besides aromatic amino acids and lipids, sugars (in particular ribose) also have significant hydrophobicity (Janado and Yano, 1985). An association of a nucleobase with ribose or deoxyribose, forming a nucleoside, would therefore have been beneficial in preventing sedimentation and thereby optimizing both photon dissipation and concentration at the air-water interface.

Simple heating of the purines through condensation/hydration cycles with ribose leads to the respective nucleoside (Fuller et al., 1972) but a route through UV irradiation was also found to be very effective (Ponnamperuma et al., 1963).

Like for the purines, a UV-C photochemical route to the synthesis of the two and three carbon sugars glycolaldehyde and glycer aldehyde and the one carbon sugar formaldehyde, necessary for the synthesis of ribose, utilizing hydrogen cyanide (HCN) and an electron donor, has been found (Ritson and Sutherland, 2012). There exists another route to the sugars through UV-C photochemical polymerization of formaldehyde in an aqueous solution with an inorganic base, the so-called “formose reaction”, which is more specific for the reduced sugar pentaerythritol than the equivalent thermal formose reaction (Shigemasa et al., 1977). However, ribose, like the purines, may also have been structured autocatalytically through photon dissipation in the gas phase from HCN and formaldehyde (or other gases), but at shorter wavelengths available in the upper atmosphere since ribose has it’s own inherent conical intersection (∼ 170 nm − 7.4 eV) and is transparent to longer wavelengths (Tuna et al., 2016). Indeed, experiments using an H_2_ discharge lamp providing Lyman α photons at 122 nm with a tail including an H_2_ recombination line at approximately 160 nm have shown that irradiation of mixtures of H_2_O, CH_3_OH, NH_3_ at low temperatures (simulating grains surrounded by icy mantles containing these substances) produced ribose and related molecules in high yield (3.5% by mass) leading the authors to suspect an unidentified autocatalytic process (Meinert et al., 2016).

### 4.3 Microscopic dissipative structuring of single strand RNA or DNA

A further important step in the evolution of life was the polymerization of the nucleosides into single strand RNA or DNA. There are both thermodynamic and non-equilibrium thermodynamic incentives for polymerization; greater stability against hydrolysis, and the provision of a scaffolding to allow dissipation fomenting molecules to attach themselves to RNA or DNA, respectively. For example, antenna type molecules which, through resonant energy transfer, could employ the conical intersections of the acceptor nucleic acid molecules to rapidly dissipate their photon- induced electronic excitation energy into heat (Michaelian, 2011; Michaelian, 2016), or, hydrophobic molecules which could have trapped the polymers at the ocean surface, exposing them to maximum irradiation. In this section I show how these thermodynamic principles based on dissipation could have led to the autocatalytic production and proliferation of single strand RNA or DNA given the existence of nucleobases, ribose, inorganic phosphates, and a UV-C light flux in the ocean surface environment.

A possible photochemical reaction scheme for the template directed autocatalytic production of single strand DNA or RNA polymers of length, say, 10 nucleotides is;

(1a) γ_260_ + HCN + H_2_O → CN_2_H_2_ + …

(1b) 3CN_2_H_2_ + 3PO_4_^3−^ + N_s_ → N_t_* + 3H_2_O + …

(1c) 10N_t_* + Mg^2+^ + ssDNA_10_ → dsDNA_10_ + Mg^2+^

In reaction (1a), a 260 nm UV-C photon interacts with hydrogen cyanide in an aqueous solution to produce cyanamide CN_2_H_2_ or dicyandiamide C_2_N_4_H_4_ (Schimpl et al., 1964). In (1b) cyanamide, or dicyandiamide, act as condensing reagents three successive times, phosphorylating a nucleoside N_s_ into an activated nucleotide N_t_* (Yamagata et al., 1979; Yamagata et al., 1981). In (1c) 10 activated nucleotides interact with a template single strand DNA, ssDNA_10_, over night at somewhat colder ocean surface temperatures, utilizing Mg^2+^ ions as polymerization catalysts to form double strand DNA, dsDNA_10_ (Szostak, 2001).

Alternatively to (1a) and (1b), phosphorylation of the nucleosides may have occurred thermally in an aqueous mixture of urea with inorganic basic or neutral phosphates such as hydroxylapatite Ca_5_(OH)(PO_4_)_3_ at temperatures between 65 and 100 °C (Lohrmann and Orgel, 1971).

Finally, McReynolds (1971) has shown that UV-C induced extension, with predominance of the 3′-5′ normal linkage, is possible starting with nucleotides even without activation in an aqueous environment;

10γ_260_ + 10N_t_ + Mg^2+^ → ssDNA_10_ + Mg^2+^

The final step common to all schemes is the UV-C induced denaturing of double strand DNA (Michaelian and Santillán Padilla, 2014);

γ_260_ + dsDNA_10_ → 2ssDNA_10_.

Here, the dissipation of the UV-C excitation energy into local heat, and the puckering of the C_2_ atom (adenine) or the C_6_ atom (guanine), and the out-of-plane distortion of the NH_2_ groups of the excited base (adenine, guanine, and cytosine) needed to reach the conical intersection (Nachtigallová et al., 2010; Plasser et al., 2014) will break hydrogen bonds allowing the double helix to denature into two single strands, ssDNA_10_ (Michaelian, 2011; Michaelian, 2016). The breaking of hydrogen bonds could also be initiated by photon-induced deprotonation of DNA. Denaturing would have occurred preferentially during the late afternoon when ocean surface temperatures were warmest.

The overall photochemical reaction is thus;

31γ_260_ + 30PO_4_^3−^ + 10N_s_ + ssDNA_10_ → 2ssDNA_10_

which is just an autocatalytic template directed photochemical reaction of the form A + B → 2B for the production of single strand DNA, with A = 31γ_260_ + 30PO_4_^3−^ + 10N_s_ and B = ssDNA_10_. The single strand DNA, ssDNA_10_, acting as a template makes the overall photochemical reaction autocatalytic.

### 4.4 Dissipative proliferation of the fundamental molecules

Following Prigogine’s non-equilibrium thermodynamic analysis of autocatalytic chemical reactions (Prigogine, 1967), and assuming the Glansdorff-Prigogine evolutionary criterion, Equation (8), I have shown (Michaelian, 2013) that, if under the imposition of the solar photon potential, and given a constant supply of reactants and a constant sink (dispersal) of the products, a photochemical route can be found to the production of a pigment molecule (such as the examples given above), and if that pigment molecule is efficient at dissipating the same photon potential that was required to produce it, then a process similar to that of the autocatalytic chemical reaction would occur, only that besides a chemical potential, a photochemical potential would also be dissipated. In a manner analogous to what happens with the product catalyst in a chemical autocatalytic reaction, the concentration of the pigment in the photochemical autocatalytic reaction at the thermodynamic stationary state would become many orders of magnitude larger than what would be expected under near equilibrium conditions, or where the pigment did not act as a catalyst for photon dissipation or its own production. The derivation shows that the greater the efficacy of the particular pigment in dissipating the solar photon potential, the greater the amount of that pigment in the stationary state that would be expected and the greater the global entropy production of the process (Michaelian, 2013). This is the dissipation-replication relation alluded to above which explains life’s perceived “vitality”.

### 4.5 Evolution

Any molecular association or novelty which increased its entropy production under a UV-C flux would therefore be proliferated (differentially selected) through this dissipation-replication relation. As an example, the 3-base pair stereochemical coding in RNA or DNA for tryptophan (Yarus et al., 2009), another UV-C pigment (see Fig. 1), may have occurred because tryptophan could have “utilized” (in a non-equilibrium thermodynamic sense) the conical intersection of RNA or DNA to rapidly dissipate its UV-C photon-induced electronic excitation energy; the complex dissipating more efficiently than the component molecules acting independently. In this way, the RNA or DNA codon with affinity to an antenna molecule such as tryptophan could also be considered as a microscopic dissipative structure created during the Archean that has persisted, and is even utilized to this day, even though the external generalized chemical potential that created and proliferated it (the UV-C photon potential) no longer exists at Earth’s surface. RNA and DNA thus eventually took on the new role of information storage, with the stored information invariably associated with the dissipation of an externally imposed generalized chemical potential at one time existent in the organisms environment (Mejía and Michaelian, 2017).

It is thus possible to envision increases in complexity concomitant with increases in dissipation as well as nested sets of autocatalytic photochemical and chemical reactions occurring, each feeding off a more fundamental photochemical reaction by using its products and the generalized chemical potential generated (e.g. heat gradients or chemical potential). Instructive examples of this were given above in the codification of tryptophan in RNA or DNA, and the photochemical production, starting from HCN, of single strand RNA or DNA. Such ideas of nested sets of autocatalytic chemical reactions giving rise to complex life are not new (e.g. Eigen, 1979; Kauffman, 1986), however, here we have identified a most important missing component; microscopic dissipative structuring and dissipative proliferation driven by the external UV-C/UV-B photon potential of the Archean.

Today, Earth's biosphere dissipates into heat a large number of UV and visible solar photons arriving at Earth's surface by utilizing an enormous quantity and variety of organic pigments, of which chlorophyll is just one notable case. For example, the carotenoids are known acceptor quencher molecules for the rapid dissipation (within 200 ps) of excited chlorophyll donor molecules, the two pigments being held close enough together for resonant energy transfer in high-light inducible proteins (Hilps) which are the cyanobacterial predecessors of the light harvesting complexes (LHC) found in plants (Staleva et al., 2015). The carotenoid-chlorophyll dissipative system may thus be a modern analog of the RNA/DNA-tryptophan dissipative system of the Archean discussed above.

Even with such a formidable array of evolved pigment complexes, the wavelength integrated flux of photons from the Sun at Earth's surface is so copious (∼10^22^ m^−2^ s^−1^) that only a portion of the solar photons arriving at Earth's surface are absorbed and dissipated by today's evolved pigments in plants, diatoms and cyanobacteria (Michaelian and Simeonov, 2015). The rest of the solar photons, principally those with wavelength longer than that corresponding to the red-edge (∼700 nm), are simply reflected with practically no energy dissipation, but, however, with entropy production since diffuse reflection distributes the photon energy over a much larger solid angle of space than that corresponding to the incident beam (Michaelian, 2012).

There appears to be no better or succinct description of biotic-abiotic co-evolution of the biosphere than the observation that the photon dissipation efficacy of Earth in its solar environment has increased ever since the formation of Earth in the Hadean. This evolution has incurred;

1. the microscopic dissipative structuring of pigments
2. a greater photon absorption cross section per pigment size, for example by increasing molecular electric dipole moments,
3. faster non-radiative dissipation of the photon-induced electronic excitation energy of pigments through evolution of molecular conical intersections,
4. quenching of the fluorescent and phosphorescent radiative decay channels, through, for example, evolving complexes of molecules practicing resonant energy transfer to conical intersections,
5. quenching of electron or proton transfer reactions, except where required for specific dissipative structuring processes, e.g. nucleobase structuring and photosynthesis,
6. stability against photochemical reactions and charge transfer reactions which could destroy the pigment,
7. new pigments absorbing at different wavelengths by extending conjugation in a molecule, thereby covering ever more completely the solar spectrum at Earth’s surface,
8. processes that exude pigments into the environment to promote dissipation over a greater part of the solar spectrum, for example, oxygenic photosynthesis leading to oxygen and ozone in the environment,
9. mechanisms for promoting the dispersal of pigments over ever more of Earth's sunlit surface and into new inhospitable environments, through, for example, the evolution of the cell, insects and other animals required for dispersing nutrients and seeds; a process catalyzed by the exudation of oxygen into the environment (point 8),
10. greater coupling of photon dissipation in organic pigments to other biotic and even abiotic irreversible processes, e.g. the water cycle, winds and ocean currents,
11. transparency of Earth's atmosphere to the most intense high energy part of the solar spectrum so that photons can arrive at Earth’s surface where they can be best intercepted by complex organic pigments in liquid water and thereby dissipated most efficiently.

The complexification of material into mobile abiotic or biotic mechanisms (organisms) for transporting the pigments (and the nutrients required for their proliferation) into regions where sunlight exists but where nutrient content is low (point 9) provides a thermodynamic explanation for the proliferation of animals and the water cycle (Michaelian, 2012). Animals, particularly insects, provided for the dispersal of nutrients, particularly phosphate, required for pigments and their support structures over continental land masses beginning about 500 million years ago. Humans appear to have had an important role in fomenting the amount of carbon in the carbon-cycle, phosphate in the phosphate-cycle, and water in the water-cycle, and the future may include an extension of this thermodynamic function to other planets, and even an alternative role; that of diverting sunlight into regions of high organic nutrient content. Such a re-direction of generalized thermodynamic forces, rather than fomenting flows, may be a useful thermodynamic definition of an “intelligent” animal.

## 6 Discussion and conclusions

The physical, chemical, optical, and electronic properties of RNA and DNA and the other fundamental molecules of life, as well as known photochemical routes to their production, suggest that these molecules were once microscopic dissipative structures that originated and proliferated over the ocean surface under an non-equilibrium thermodynamic imperative to dissipate the prevailing Archean solar UV-C/UV-B photon flux. Microscopic dissipative structuring involves internal degrees of freedom of the pigment molecules such as photon-induced isomerizations, tautomerizations, and activation. Their proliferation to far beyond equilibrium concentrations can be understood in terms of non-linear, non-equilibrium thermodynamic principles directing autocatalytic photochemical reactions in which these pigments catalyze the dissipation of the same thermodynamic potential (the solar photon flux) that produced them.

Complexification of life would have been driven by differential proliferation of complexes which increase photon dissipation efficacy most through this dissipation-replication relation. Distinct complexes could, of course, eventually have come into competition for limited resources but those complexes “surviving” would not have been selected based on an ill-defined “fitness function” but rather based on their contribution to the efficacy of entropy production through differential dissipative proliferation.

Evolution has been, and still is, primarily concerned with increasing the rate of photon dissipation in the biosphere. Photon dissipation continues to this day as the most important thermodynamic work performed by life, but now with pigments operating in the near UV and visible regions of the solar spectrum where the solar light is most intense, while dissipation of the UV-B and UV-C regions has been mostly delegated to ozone derived from oxygen, which should be considered as a pigment exuded by oxygenic photosynthetic organisms into the environment. The production of pigments today to far beyond their expected equilibrium concentrations is similarly driven, but now indirectly, through complex biosynthetic pathways employing autocatalytic photochemical reactions operating in the near UV and visible.

Microscopic self-organization and proliferation through dissipation and the persistence of nanoscale structuring at the origin of life would not be unique to Earth but should have occurred wherever there once existed UV-C or UV-B light and organic elements; in protoplanetary systems, on planets, on asteroids, on comets, and even in the galactic gas and dust clouds. Organic pigments are, in fact, found ubiquitously throughout the cosmos where UV light is available (Michaelian and Simeonov, 2017).

Organic pigments on Titan catalyzing the methane rain cycle, or the sulfur containing pigments floating in the clouds of Venus catalyzing the great southern and northern vortices, may be examples of microscopic dissipative structuring giving rise to distinct dissipative “biospheres” on other planets. The cosmos should be teaming with such ecosystems based on pigment “self-organization” through microscopic dissipative structuring and dissipative proliferation under the photon potential of the local star.

## Declarations

### Author contribution statement

Karo Michaelian: Conceived and designed the experiments; Performed the experiments; Analyzed and interpreted the data; Contributed reagents, materials, analysis tools or data; Wrote the paper.

### Competing interest statement

The author declares no conflict of interest.

### Funding statement

This work was supported by the Dirección General de Asuntos del Personel Academico, DGAPA-UNAM, project number IN102316.

### Additional information

No additional information is available for this paper.
